# A Comparative Pharmacokinetics Study of Orally and Intranasally Administered 8-Nitro-1,3-benzothiazin-4-one (BTZ043) Amorphous Drug Nanoparticles

**DOI:** 10.1021/acsptsci.4c00558

**Published:** 2024-11-09

**Authors:** Feng Li, Franziska Marwitz, David Rudolph, Wiebke Gauda, Michaela Cohrs, Paul Robert Neumann, Henrike Lucas, Julia Kollan, Ammar Tahir, Dominik Schwudke, Claus Feldmann, Gabriela Hädrich, Lea Ann Dailey

**Affiliations:** † Department of Pharmaceutical Sciences, Division of Pharmaceutical Technology and Biopharmaceutics, University of Vienna, Josef-Holaubek-Platz 2, 1090 Vienna, Austria; ‡ Division of Bioanalytical Chemistry, Research Center Borstel, Leibniz Lung Center, Borstel 23845, Germany; § Institute of Inorganic Chemistry, Karlsruhe Institute of Technology (KIT), Engesserstraße 15, Karlsruhe 76131, Germany; ∥ Laboratory for General Biochemistry and Physical Pharmacy, Ghent University, 9000 Gent, Belgium; ⊥ Department of Pharmaceutical Technology and Biopharmaceutics, Institute of Pharmacy, Martin Luther University Halle-Wittenberg, Halle (Saale) 06120, Germany; # Department of Pharmaceutical Sciences, Division of Pharmacognosy, University of Vienna, Josef-Holaubek-Platz 2, 1090 Vienna, Austria; ∇ Vienna Doctoral School of Pharmaceutical, Nutritional and Sport Sciences (PhaNuSpo), University of Vienna, Josef-Holaubek-Platz 2, 1090 Vienna, Austria; ○ Thematic Translational Unit Tuberculosis, German Center for Infection Research (DZIF), Borstel 23845, Germany; ◆ German Center for Lung Research (DZL), Airway Research Center North (ARCN), Research Center Borstel, Leibniz Lung Center, Borstel 23845, Germany; ¶ Kiel Nano, Surface and Interface Sciences (KiNSIS), Kiel University, Kiel 24118, Germany

**Keywords:** BTZ043, MDR-TB, amorphous drug nanoparticles, lung targeting, pharmacokinetics

## Abstract

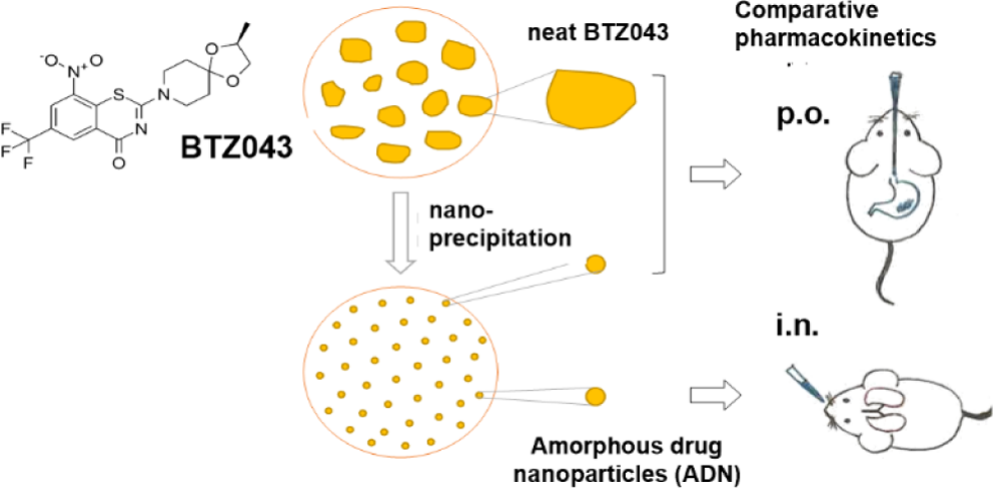

BTZ043 is an 8-nitro-1,3-benzothiazin-4-one with potency against multidrug-resistant *Mycobacterium tuberculosis*. Low solubility and hepatic metabolism are linked to poor oral bioavailability. Amorphous drug nanoparticles (ADN) were formulated to improve the bioavailability. Comparative pharmacokinetics of BTZ043 ADN following intranasal (2.5 mg kg^–1^) and oral administration (25 mg kg^–1^) in Balb/c mice was investigated using oral BTZ043 drug suspensions (neat; 25 mg kg^–1^) as a standard-of-care reference. Plasma exposure following oral ADN administration was 8-fold higher than for oral neat BTZ043. Intranasal ADN increased plasma exposure 18-fold compared to oral neat BTZ043 after dose normalization. BTZ043 was detectable in lung lining fluid following ADN administration, but not after oral neat BTZ043 dosing. BTZ043 was cleared faster from the lung and plasma following intranasal administration with a shorter time above the minimum inhibitory concentration (MIC) compared to oral ADN. Since time > MIC is reported to drive activity, oral ADN may represent a promising delivery strategy for BTZ043.

The research and development of new or repurposed drugs have become the focus of the treatment of multidrug-resistant (MDR) tuberculosis (TB), which remains a significant challenge.^1−3^ Presently, MDR-TB is difficult to treat because there are limited options for second- or third-line therapeutics and these are associated with significant side effects, which leads to poor patient compliance.^4^ These issues are mostly due to the lower efficacy of the drugs and a long treatment duration of at least two years. New strategies aiming to achieve a therapeutic level in the lesions of TB infection are urgently needed, and ideally, such a therapeutic scheme would achieve concentrations above the minimal inhibition concentration (MIC) in the granulomas. Compared with the known second-line anti-TB drugs that require large oral doses, the development of new antibiotics with higher antibacterial activity is one aim of the current research. Drugs with novel anti-TB targets or increased granuloma penetration are effective ways to enhance antimicrobial efficiency.^5−7^

1,3-Benzothiazin-4-ones (BTZs) belong to a new class of antimycobacterial agents for MDR-TB treatment first reported by Makarov et al.^8^ BTZs specifically target the cysteine residue (Cys387) in the active site of decaprenylphosphoryl-β-d-ribose 2′-epimerase (DprE1) in *Mycobacterium tuberculosis*. DprE1 is confirmed to be highly conserved and can reduce the nitro group of BTZs to nitroso to form an irreversible hemithiol adduct.^9−11^ Deactivating the function of DprE1 blocks the arabinan synthesis of the cell wall and therefore inhibits the growth of *M. tuberculosis*.^12^ BTZ043 is the leading compound in this class which showed extremely high antibacterial activity with the in vitro MIC of 0.001 and 0.004 μg mL^–1^ against *M. tuberculosis* H37Rv and *Mycobacterium smegmatis*, respectively.^8^ Drug susceptibility testing showed that BTZ043 is effective in pan-sensitive (*n* > 20), monodrug-resistant (*n* > 10), and MDR- and extremely drug-resistant (XDR-) clinical *M. tuberculosis* isolate strains.^8^ In vitro drug combination studies showed that BTZ043 has no antagonistic effect with other anti-TB antibiotics that also inhibit the synthesis of *M. tuberculosis* cell wall components including current first-line drugs (isoniazid and ethambutol), pretomanid, and meropenem. No antagonistic effect was found with drugs that target bacterial DNA-dependent RNA polymerase (rifampicin), DNA gyrase enzyme (moxifloxacin), and mycobacterial ATP synthase (bedaquiline (BDQ)), suggesting a good compatibility in combination therapy.^13^

BTZ043 is moderately hydrophobic with a log P of 2.84^8^ and the lipophilicity promotes the accumulation of BTZ043 in the foamy macrophage layer surrounding granulomas further increasing the concentration gradient and helping with the penetration into the caseous necrosis center via passive diffusion.^14^ This was demonstrated in studies with NOS2-deficient mice, which mimic the structure of human pulmonary TB-infected lesions, where BTZ043 had a uniform distribution in the tissue and sufficient penetration into the granulomas.^15^ However, the efficacy after oral administration has been disappointing. Even with a low MIC and good permeability, the sterilization ability of BTZ043 (37.5 mg kg^–1^) in chronic TB infection mouse models is still lower than that of isoniazid (INH, 25 mg kg^–1^) and RIF (10 mg kg^–1^). Increasing the dosage to 300 mg kg^–1^, BTZ043 showed similar efficacy as RIF in the lung tissue but was less effective in the spleen when compared to INH.^12^

An oral bioavailability of 29.5% was reported in Sprague–Dawley rats with a single dose of 5 mg kg^–1^^16^ which is hypothesized to be due to a combination of low aqueous solubility and high metabolism rate in the rodent plasma.^17,18^ Lung-targeted drug delivery has been shown to achieve fast onset of action and high local drug concentrations in many other diseases. However, one of the challenging aspects of lung drug delivery is achieving a sufficient dose within a limited volume of the dosage form. Further, the delivery of poorly soluble micronized drug powders to the lungs has been associated with safety issues and poor local tolerability.^19^

Amorphous drug nanoparticles (ADN) are solid particle suspensions in which a drug (especially BCS class II) is precipitated into amorphous nanoparticles or dispersed in a nanosized carrier.^20^ The relatively disordered amorphous structure can increase the active surface area of the particles, thereby increasing the solubility and dissolution rate of the drug. According to the Noyes–Whitney equation (eq 1), the increase in the effective surface area of the ADNs is directly proportional to an accelerated rate of dissolution^21^
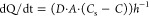
1where (d*Q*/d*t*) represents the dissolution rate, (*D*) diffusion coefficient, (*h*) thickness of unstirred water layer at the solid surface, (*A*) specific surface area of the solid, (*C*_s_) saturation concentration, and (*C*) concentration in bulk solution. In addition to the increased surface area, the metastable amorphous state can also promote the formation of supersaturated solutions. In some cases, the solubility can be increased theoretically as much as 10–1600 times in the supersaturated state compared to the crystalline nanoparticles.^22^ Whether this solubility advantage is in practice as large as predicted depends on the equilibrium conditions and true thermodynamic considerations,^21,22^ Yang et al. reported that amorphous nanostructured aggregates have a similar dissolution rate as wet-milled drugs but with a 4.7 times higher supersaturation.^23^

With regard to pulmonary administration, the tailored dissolution kinetics of an inhaled BTZ043 ADN formulation would ideally be slow enough to prevent immediate drug permeation across the air-blood barrier, thereby maintaining a high lung retention of the drug. At the same time, the BTZ043 must dissolve rapidly enough to prevent the accumulation of poorly soluble drug particles in the lung. Indirect evidence for this phenomenon was recently reported by Rudolph et al., whereby BTZ043 ADNs (99% BTZ043 with <1% sodium dodecyl sulfate) were tested for antitubercular efficacy in a mouse model.^24^ The ADNs exhibited nearly twice the amount of dissolved drug after 24 h in vitro incubation (19.9 ± 1.3%) compared to the micron-sized neat BTZ043 (9.5 ± 1.3%),^24^ demonstrating a modified drug dissolution profile. In a C3HeB/FeJ mouse model of infection with the *M. tuberculosis* H37Rv strain, intranasally administered BTZ043 ADNs achieved a 50% higher reduction in lung burden compared to BTZ043 in solution form, while showing a similar efficacy as the solubilized drug in the spleen (dose: 6.5 mg kg^–1^ instilled intranasally every second day for 2 weeks).^24^ These results suggest that the BTZ043 ADN were retained in the lung longer than solubilized BTZ043, achieving better antibacterial efficacy in this organ, while still able to permeate across the air-blood barrier in sufficient concentrations to achieve activity in the spleen.

In the current study, the in vivo pharmacokinetics (PK) of intranasally administered BTZ043 ADNs were compared with oral administration of the ADN formulation in healthy Balb/c mice. Further, both study groups were compared with an orally dosed neat BTZ043 drug suspension, which was used as a standard-of-care comparator. The two-fold purpose of this study was to assess the relative lung and plasma concentrations achieved via the oral versus intranasal administration route, while also determining whether the ADN formulation improved the PK profiles compared to oral administration of the neat drug. We hypothesized that orally administered ADN would dissolve more rapidly than neat drug, thereby increasing plasma concentrations and consequently drug concentrations in the lung. Second, intranasal administration of ADN would achieve higher lung concentrations of BTZ043 compared to oral administration (Figure 1A).

**Figure 1:**
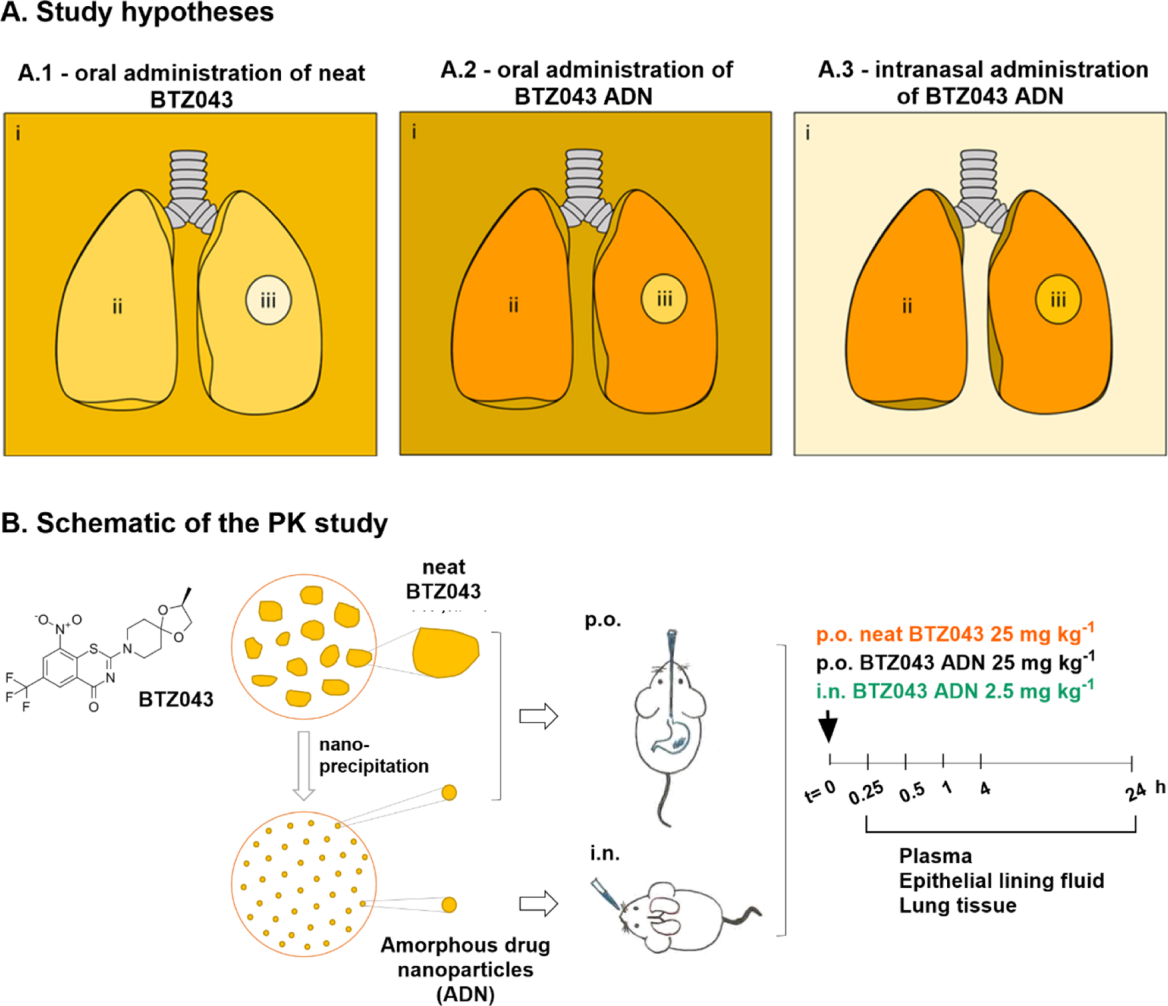
(A) Hypotheses of drug distribution after oral administration of neat drug (A.1), oral administration of BTZ043 ADN (A.2), and intranasal administration of BTZ043 ADN (A.3). Three different compartments are marked as (i) plasma, (ii) lung, and (iii) granulomas. The darker yellow color indicates the higher hypothesized concentration of BTZ043. (B) Schematic of the PK study; p.o., per os or by mouth; i.n., intranasal administration.

## Results and Discussion

### Sample Properties: Neat BTZ043 vs BTZ043 ADN

**Figure 2:**
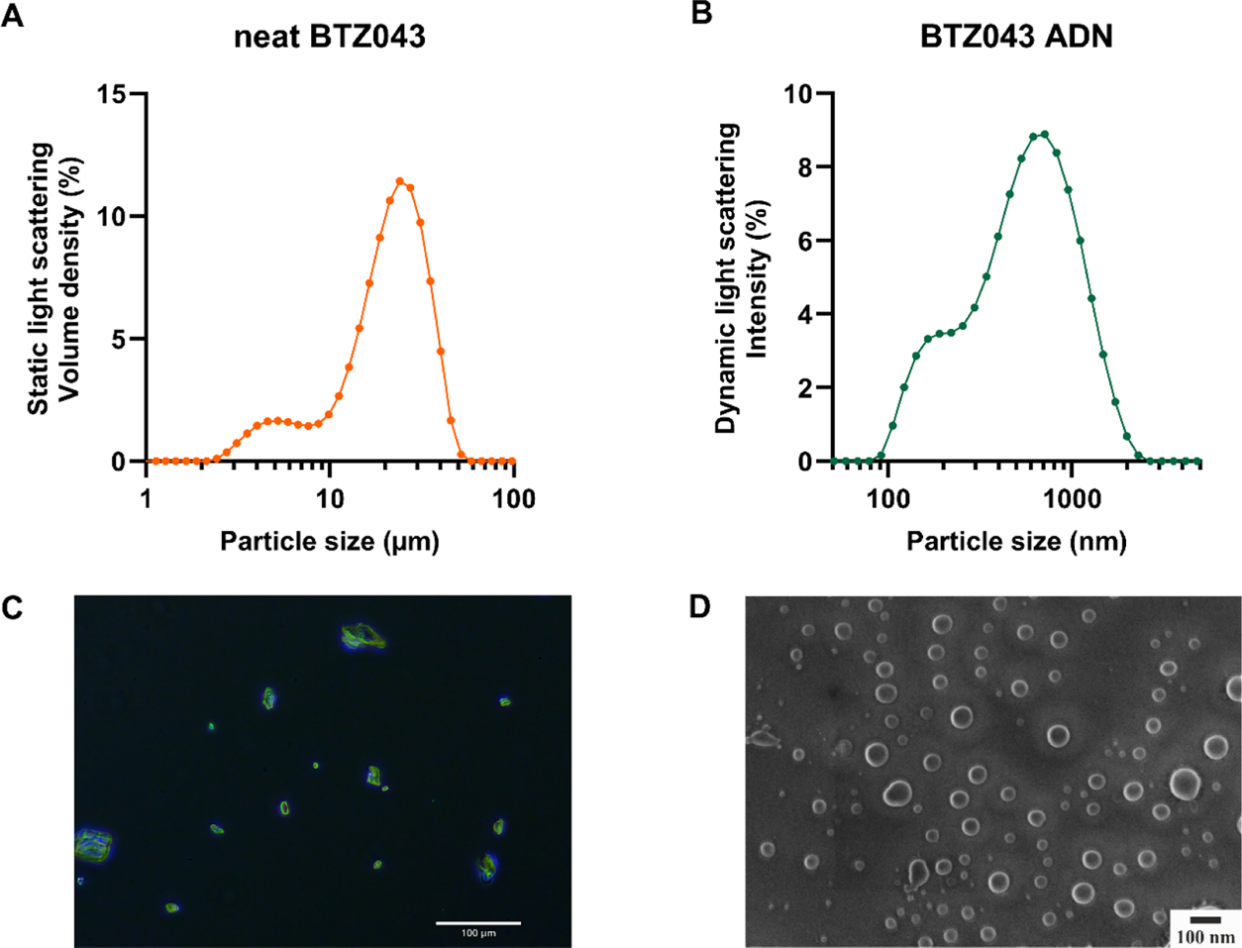
(A) Particle size distribution of neat BTZ043 powders dispersed in water containing 2.5% Kolliphor HS15 (1 mg mL^–1^) measured by static light scattering (μm) and (B) dynamic light scattering (DLS) (nm) of BTZ043 ADN formulations (10 times dilution in phosphate-buffered saline (PBS)) on the day of the in vivo study. (C) Light microscopy images of neat BTZ043 dispersion (scale bar 100 μm). (D) Scanning electron microscope image of BTZ043 ADN (scale bar = 100 nm). Image reproduced from Rudolph et al.^24^ Copyright 2023 American Chemical Society.

Neat BTZ043 was composed of a crystalline powder with irregularly shaped particulates ranging roughly from 5 to 40 μm in diameter (Figure 2). Static light scattering measurements showed that the median particle size (D50) was 23 μm with D10 and D90 values of 8 and 38 μm, respectively. D10 and D90 are defined as the particle diameters at which 10% and 90% of the particles in the sample are smaller in size.^25^ Suspensions produced from the neat BTZ043 powder were included in this study as a proxy for a typical standard-of-care product in either tablet or capsule form. It was hypothesized that slow gastrointestinal dissolution of the larger neat drug particulates represents the main rate-limiting step for in vivo BTZ043 absorption.

The BTZ043 ADNs were prepared with a drug concentration of 4.2 mg mL^–1^ and drug loading of >99%. SEM images of the freshly prepared sample (Figure 2D) show a primary particle size of ∼60 nm,^24^ while dynamic light scattering measurements performed in the administration vehicle, phosphate-buffered saline (PBS) on the day of administration (after storage of approximately 1–2 weeks) exhibited hydrodynamic diameters of 439 ± 4 nm with polydispersity indices of 0.31–0.34 (Figure 2B), suggesting that a small to moderate amount of ADN aggregation was occurring in the vehicle. However, since the BTZ043 ADN aggregates were still substantially smaller than the neat BTZ043 particulates with a correspondingly larger surface area for more rapid dissolution, this observation was noted but not considered to be a confounding factor for the current study.

### Impact of BTZ043 Formulation on Calu-3 Apparent Permeability

**Figure 3:**
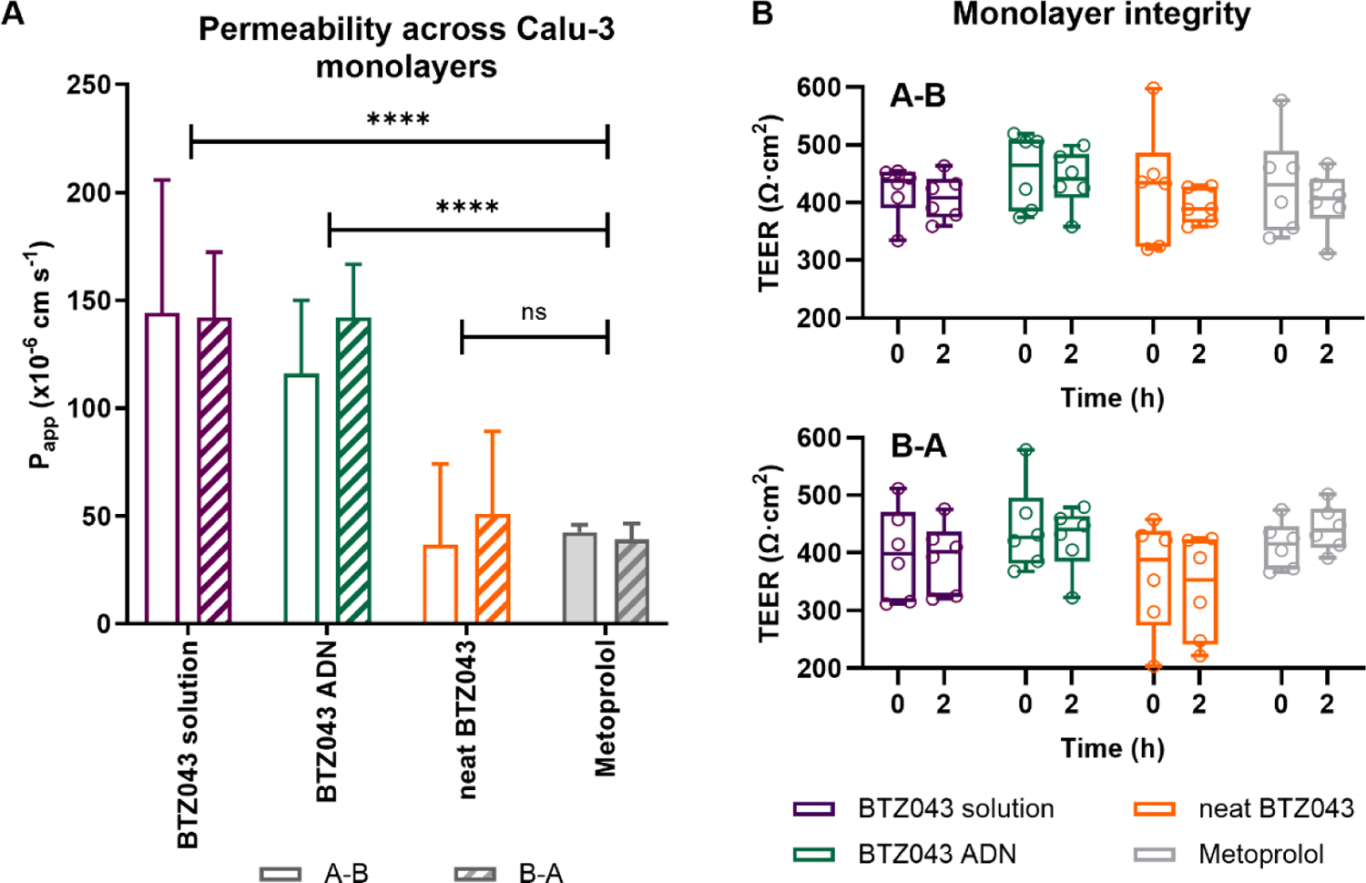
(A) Apparent permeability of BTZ043 solution (Hanks’ balanced salt solution (HBSS) with 0.05% DMSO), ADNs, and neat drug suspension across Calu-3 cell monolayers. (B) Box and whisker plots of the transepithelial electrical resistance (TEER; Ω·cm^2^) before and after permeability studies in the apical to basolateral (A-B; top) and basolateral to apical (B-A; bottom) direction. No significant drop in TEER was observed before and after experiments. Values represent the mean ± standard deviation of *n* = 6 independent experiments. ****p < 0.0001, ns = nonsignificant.

In a preprint, Treu et al.^14^ report apparent permeability (*P*_app_) values of ∼10 × 10^–6^ cm s^–1^ in both A to B and B to A directions for BTZ043 solutions (containing dimethyl sulfoxide (DMSO)) in a Caco-2 monolayer model of the intestinal epithelium. This data indicates that the primary mechanism of transport is passive diffusion across the cell membrane. In the current study (Figure 3A), the apparent permeability across monolayers of the lung cell line, Calu-3, was measured. *P*_app_ values of the BTZ043 neat drug suspension were 37 × 10^–6^ and 51 × 10^–6^ cm s^–1^ for A to B and B to A directions, respectively. This value was higher than the reported values for BTZ043 across Caco-2 cell monolayers and comparable studies in Calu-3 cell monolayers under an air–liquid interface,^26^ which may be due to the lower transepithelial epithelial resistance (TEER) value than the references (Figure 3B).

It could be confirmed that solubilized BTZ043 has a significantly higher *P*_app_ value than metoprolol (*p* < 0.0001), a drug commonly used to distinguish high from low permeability compounds.^26,27^ Furthermore, we observed no significant differences in directional transport, confirming that BTZ043 traversed the epithelial barrier primarily via passive diffusion through the cell membrane.^27,28^ BTZ043 ADN formulations also showed a significantly higher *P*_app_ compared to neat BTZ043 suspensions (*p* < 0.0001), likely due to differences in dissolution rate. Interestingly, the *P*_app_ of BTZ043 ADN formulations was similar to that of the dissolved BTZ043 (*p* = 0.39). It is important to consider that the concentration used for permeability studies was 10 μM (4.3 μg mL^–1^), which was higher than the solubility (1 μg mL^–1^) reported by Xiong et al.,^29^ but below the solubility value (32 μM; 13.8 μg mL^–1^) reported by Richter et al.^30^ Thus, BTZ043 ADN may have undergone rapid dissolution in the transport medium, while the dissolution rate of the neat BTZ043 suspension was slower, reducing the measured *P*_app_.

### Comparative Pharmacokinetics

The primary aim of the current study was to underpin the results of Rudolph et al. by providing quantitative data on BTZ043 exposure in the lung and plasma following intranasal and oral administration of BTZ043 ADN.^24^ It should be noted that i.n. administration was used in the Rudolph et al.^24^ study as a less invasive method for intrapulmonary administration because of its suitability for multiple administrations in the mouse model.^24,31−33^ The current study used a similar administration protocol to make the results more comparable. As outlined in Figure 1, two major hypotheses were investigated in this study: (1) Intranasal administration of BTZ043 ADN should result in higher local lung concentrations compared to oral administration of ADN or neat drug due to direct delivery of the ADN to the lung and (2) oral administration of ADN should improve both plasma and lung bioavailability compared to the neat drug due to a more rapid dissolution profile in the gastrointestinal tract. A dose of 25 mg kg^–1^ was chosen for the oral administration groups to benchmark BTZ043 PK profiles with previously published studies.^8,14^ Limited by volume and ADN stability at high concentrations, a 10-fold lower dose (2.5 mg kg^–1^) was chosen for intranasal administration.

### BTZ043 Quantification in Lavaged Lung Tissue

**Figure 4:**
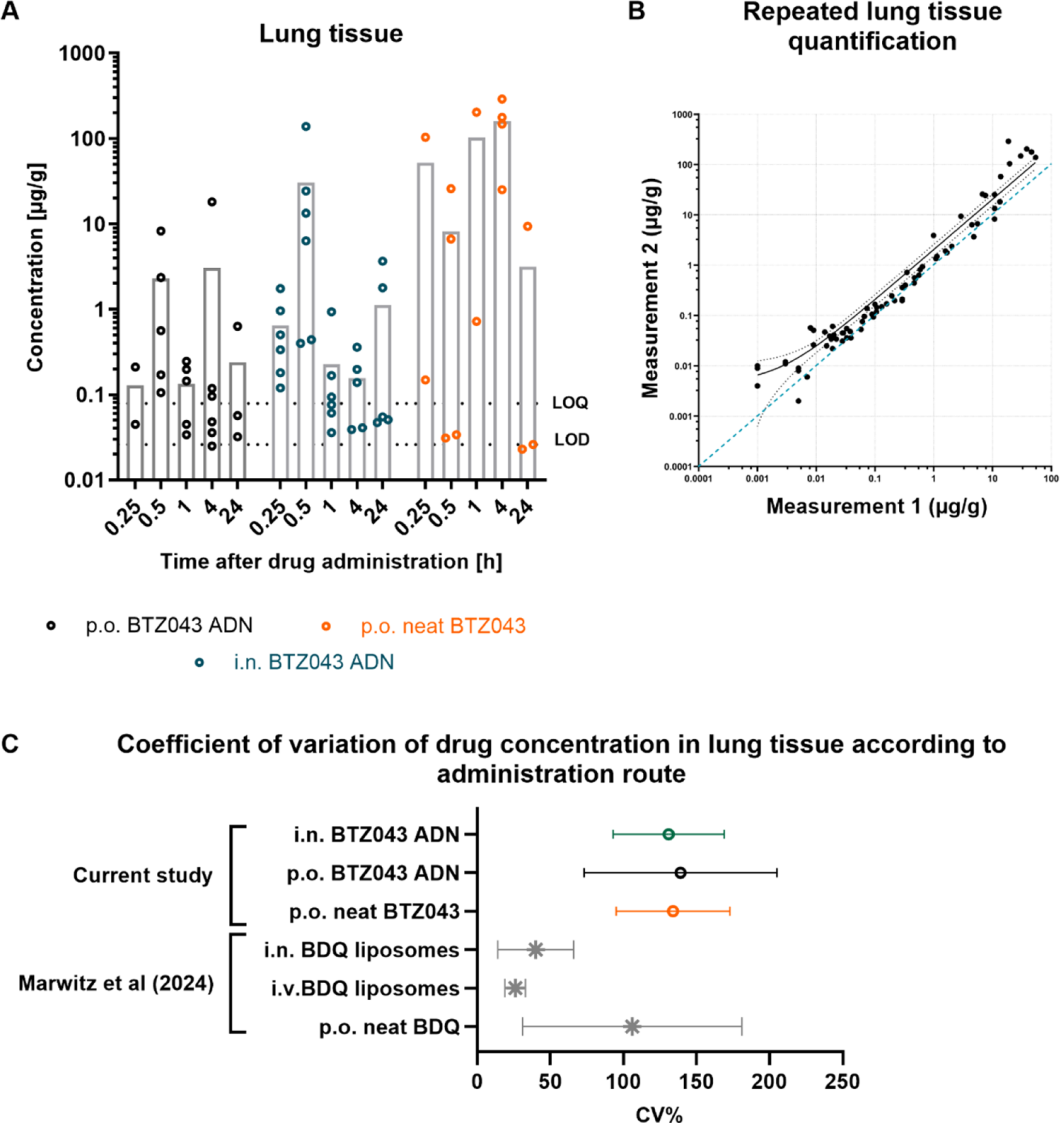
(A) BTZ043 concentrations in lung tissue samples from measurement #2 at time points 0.25, 0.5, 1, 4, and 24 h postadministration (gray bars indicate the mean value). The limit of detection (LOD) and limit of quantification (LOQ) values are depicted as dotted lines (samples with values below the LOD are not shown). (B) Lung tissue samples were quantified on two different occasions to study the reproducibility of the quantification procedure. Values from each measurement are plotted against each other. The dotted blue line denotes a 1:1 correlation. (C) Mean and standard deviations of coefficient of variation (CV%) values are shown for each administration route and BTZ043 formulation. Post hoc analysis of CV% values of BDQ measured in lung tissue from Marwitz et al.^33^ are added for comparison.

Lung exposure to a drug substance can be determined using whole lung tissue homogenates^34−36^ or as a separate analysis of tissue-bound drug and drug recovered in epithelial lining fluid (ELF).^37−41^ In this study, we chose to quantify BTZ043 in the lavaged lung tissue and ELF separately, since unbound drug in the ELF has been argued to represent the pharmacologically active fraction in the lung for many antibiotic substances.^37−41^ Interestingly, quantification of tissue-bound BTZ043 from the lavaged lung proved to be unreliable, with large variations between replicates and no consistent temporal trends (Figure 4A).

To understand whether the sample processing and measurement procedure led to a high variability, two repeated (randomized, blind) measurements were performed with samples from the lung tissue study. A comparison of the first and second measurements showed a good match between values in the intermediate concentration range but a tendency toward higher values in the upper and lower quantiles of the second measurement (Figure 4B). Despite these deviations at the ends of the spectrum, the validation study confirmed that the high variability in the measured lung tissue concentrations was not artifactual, but more likely associated with the drug distribution and tissue binding properties themselves.

To assess the inherent variability in drug recovered from lung tissue using this protocol, we employed a posthoc analysis of previously published data from Marwitz et al. which investigated the PK profile of a highly lipophilic drug, bedaquiline (BDQ).^33^ The BDQ study data is unique in that the same study design, administered doses, and sample preparation/processing protocols were used, making comparisons between the two data sets valid. Major differences between the studies included drug properties, formulation (ADN vs liposomes), the inclusion of an intravenous administration route in the BDQ study, and the time points chosen for analysis. To assess the variability of the drug in lung tissue, coefficient of variation (CV%) values were determined from the replicate concentrations at each time point. Subsequently, the mean CV% ± standard deviation from all time points was determined. The mean CV% of BDQ concentrations in lung tissue were substantially lower after i.n. and i.v. administration of 2.5 mg kg^–1^ liposomal BDQ compared to i.n. administration of 2.5 mg kg^–1^ BTZ043 ADN (Figure 4C). However, oral administration of neat suspensions of both BDQ and BTZ043 was associated with an equally high variability of the drug in lung tissue. This comparison indicates that (1) BTZ043 shows an inherently high variability in lung tissue regardless of administration route and (2) the oral administration route may be generally associated with a higher variability of drug in lung tissue. A further difference between BTZ043 and BDQ behavior was that BDQ concentrations in the lung tissue decreased consistently over time, in contrast to BTZ043 that showed no temporal trend. We therefore conclude that BTZ043 concentrations in lavaged lung tissue cannot provide reliable information about lung exposure to the drug.

### BTZ043 Quantification in ELF

**Figure 5:**
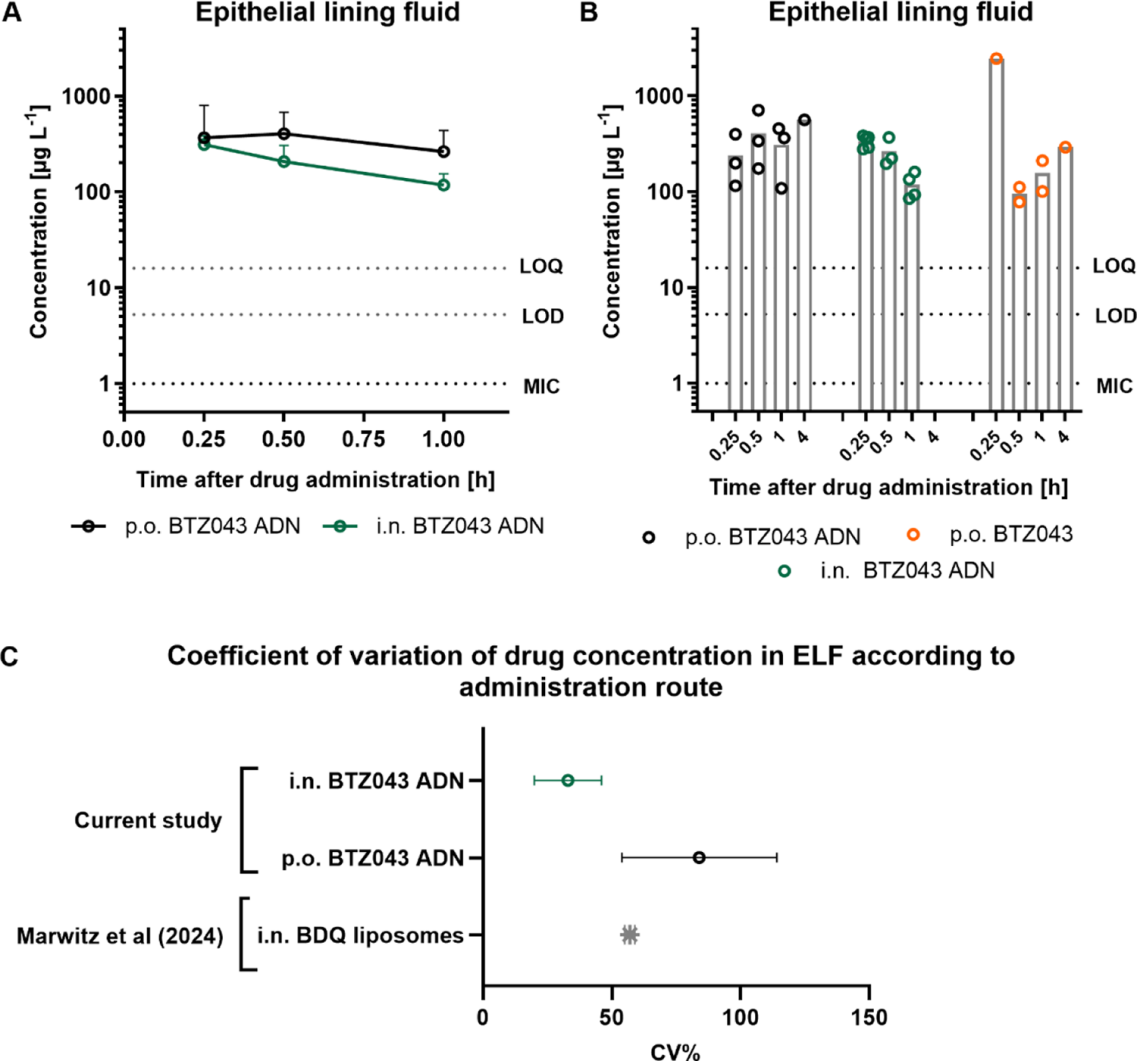
(A) PK profile of BTZ043 in ELF following i.n. and po administration of BTZ043 ADN. Values depict the mean ± standard deviation of *n* = 3–6 values per time point. (B) Individual concentrations in ELF at time points 0.25, 0.5, 1, and 4 h postadministration (gray bars indicate the mean value). The LOD and LOQ values are depicted as dotted lines (samples with values below the LOD are not shown). (C) Mean and standard deviations of CV% values are shown for each administration route and BTZ043 formulation. Post hoc analysis of CV% values of BDQ measured in ELF from Marwitz et al.^33^ are added for comparison.

In marked contrast to lung tissue, BTZ043 could be detected (minimum > three samples above LOD) in the ELF (Figure 5A,B), at least in the first hour postadministration. When detected above the LOD, the variability of BTZ043 concentration in the ELF (expressed as CV%; Figure 5C) was lower than in lung tissue and comparable to i.n. administered liposomal BDQ (46). Despite the 10-fold lower dose, i.n. administration of BTZ043 ADN achieved only marginally lower concentrations in the ELF compared to the oral administration of the ADN. Calculation of the respective AUC_0–1 h_ values in ELF resulted in 298 vs 173 μg L^–1^ h for the p.o. vs i.n. administration of BTZ043 ADN, respectively (Table 1). Normalization by dose (using eq 4) shows that i.n. administration achieves a 582% increase in drug exposure in the ELF compartment compared to oral ADN over the first hour postadministration. This short-term elevated exposure can be attributed to both the fraction of formulation which is aspirated directly into the lung following i.n. administration and the fraction of the dose that dissolves and is absorbed in the nasal cavities or GI tract. Orally administered neat drug resulted in only 1–2 animals with BTZ043 in ELF above the LOD and LOQ (Figure 5B) and was therefore not included in the analysis. The poor and uneven distribution of BTZ043 into the ELF after neat drug administration could be again reflective of the slower GI dissolution of the large crystalline drug particles and therefore lower absorption rate, especially when compared to that of the oral BTZ043 ADN treatment group.

**Table 1:** Noncompartmental PK Parameters in ELF and Plasma Calculated for Each of the Three Treatment Groups^a^

compartment	ELF			plasma		
treatment group	i.n. ADN	p.o. ADN	p.o. neat	i.n. ADN	p.o. ADN	p.o. neat
*C*_max_ (μg L^–1^)	312	407	ND	538	1101	178
*t*_max_ (h)	0.25	0.5	ND	0.25	0.5	0.25
AUC_0–1 h_ (μg L^–1^ h)	173	298	ND			
AUC_0–4 h_ (μg L^–1^ h)				448	1956	243

a ND = not determined. AUC values were calculated with GraphPad Prism software using the respective LOQ concentrations for each compartment as a baseline.

### BTZ043 Quantification in Plasma

**Figure 6:**
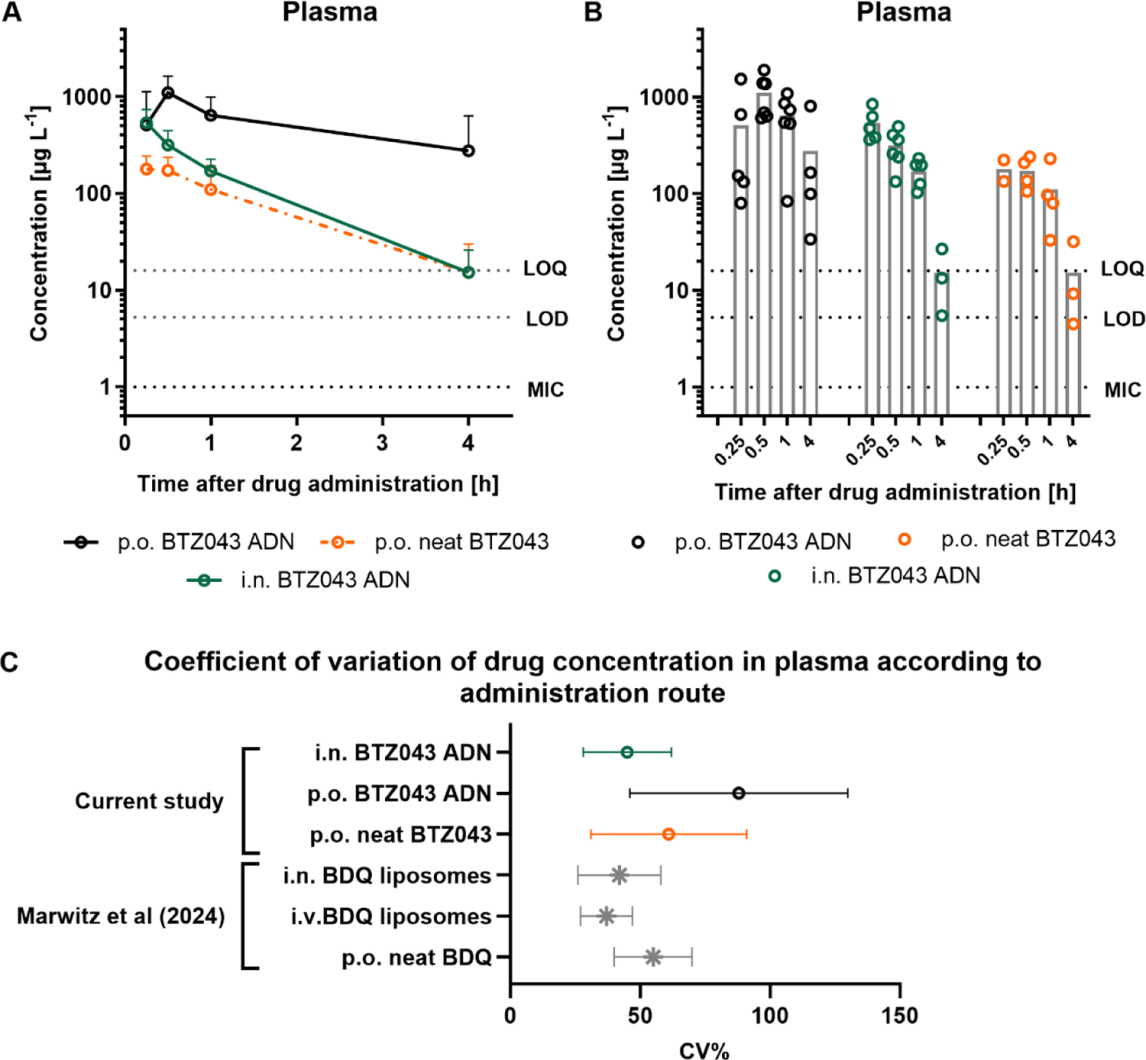
(A) PK profile of BTZ043 in plasma following i.n. and po administration of BTZ043 ADN and neat drug. Values depict the mean ± standard deviation of *n* = 3–6 values per time point. (B) Individual concentrations in plasma at time points 0.25, 0.5, 1, and 4 h postadministration (gray bars indicate the mean value). The LOD and LOQ values are depicted as dotted lines (samples with values below the LOD are not shown). (C) Mean and standard deviations of CV% values are shown for each administration route and BTZ043 formulation. Post hoc analysis of CV% values of BDQ measured in plasma from Marwitz et al.^33^ are added for comparison.

Quantification of BTZ043 in plasma revealed interesting insights into drug behavior (Figure 6). Supporting one of the original hypotheses, oral administration of BTZ043 ADN resulted in an 8-fold increase in BTZ043 systemic exposure compared to neat BTZ043 (Figure 6A), with AUC_0–4 h_ values of 1956 vs 243 μg L^–1^ h for ADN and neat drug, respectively (Table 1). While the oral ADN group showed elevated drug concentrations for nearly all animals in each time point group, neat drug administration included several replicates where drug concentration was < LOD (Figure 6B). As discussed previously, this variability likely results from different dissolution kinetics. Furthermore, access to food was not restricted prior to dosing and variable amounts of food present in the GI tract will likely influence the dissolution and absorption kinetics of BTZ043.^42^ One of the reported advantages of oral nanoformulations is a reduced variability in bioavailability between fasting and fed states,^20^ an observation which is supported by this data set.

I.n. administration of BTZ043 ADN also achieved a substantially higher systemic exposure compared to oral neat drug administration (Figure 6A and Table 1), with a surprisingly low variability in the i.n. treatment group (Figure 6C). When normalizing for dose, the i.n. administration method achieved a 2-fold increase in systemic exposure compared to oral ADN and an 18-fold increase compared to oral neat drug. However, while the systemic concentration of BTZ043 following i.n. administration decreased rapidly within the first 4 h postadministration, plasma levels remained consistently elevated for the oral ADN treatment group, which could indicate a more prolonged phase of dissolution and absorption from the GI tract, compared to a rapid dissolution and absorption from the nasal cavities and lung following i.n. administration. Treu et al.^14^ report results from oral BTZ043 dose fractionation studies in mice that indicate time > MIC (rather than *C*_max_ or AUC > MIC) as the plasma PK index most likely to drive BTZ043 activity in the mouse. Following this logic, oral administration of BTZ043 ADN may be more therapeutically advantageous, since inhalation administration of ADN may result in a faster clearance rate with a shorter time above MIC (both in the lung and systemically).

### Benchmarking BTZ043 Plasma PK Parameters to the Literature

To confirm that our reported findings are in line with reported BTZ043 PK studies in the literature, noncompartmental plasma PK parameters were compared with two other benchmark studies, Makarov et al.^8^ and Treu et al.^14^ (Table 2). Makarov et al. measured plasma PK profiles after oral administration of 25 mg kg^–1^ without disclosing information about the vehicle or any possible solubilization additives.^8^ Their measured *C*_max_, *t*_max_, and AUC values were higher than our values for both orally administered neat drug and BTZ043 ADN, but in a similar magnitude. Since the use of solubilization agents, such as DMSO or cyclodextrins, is common in PK studies, it is possible that the drug was solubilized prior to administration, which could account for such differences. A further interesting comparison with the literature is the plasma exposure data reported by Treu et al.^14^ following oral administration of 2.5 mg kg^–1^ of a nondisclosed amorphous BTZ043 formulation for five consecutive days. This is a particularly interesting comparison since the administration dose matches our i.n. ADN study group. Surprisingly similar PK parameters were reported for both studies, despite the different administration routes (i.n. vs p.o.). Importantly, both the Treu et al.^14^ data and the current study highlight the importance of formulation strategy for the improvement of the PK profile of BTZ043.

**Table 2:** Comparisons of Plasma PK Parameters of Intranasal ADN and a Nondisclosed Amorphous Drug Formulation Reported by Treu et al. Administered Using the Same Dose of 2.5 mg kg^–1^

plasma PK parameters	current study		Makarov et al.^8^	Treu et al.^14^^14^
animals	Balb/c mice (3 female, 3 male)		mice (3 female)	Balb/c mice (*n* = 3)
formulation	ADN	ADN	not described	unknown amorphous state
administration route	p.o.	i.n.	p.o.	p.o.
dose schedule	single dose, 25 mg kg^–1^	single dose, 2.5 mg kg^–1^	single dose, 25 mg kg^–1^	5 days, 2.5 mg kg^–1^
*C*_max_ (μg L^–1^)	1101	538	1923	520
*t*_max_ (h)	0.5	0.25	1	0.5
AUC (μg L^–1^ h)	1956 (0–4 h)	448 (0–4 h)	4330 (0–8 h)	666 (0–8 h)

## Conclusions

This comparative PK study contributes complementary knowledge to the growing body of literature on the bioavailability BTZ043 in a murine model. Addressing the question of whether inhalation administration of BTZ043 ADN could increase drug concentrations in the lung in a therapeutically relevant manner, we were able to show that i.n. administration led to higher BTZ043 exposure levels in both the ELF and plasma, with substantially less variation compared to oral delivery, even when administered at a 10-fold lower dose. However, BTZ043 clearance kinetics appeared to also be more rapid following i.n. administration of ADN, meaning that the time > MIC in both plasma and ELF was shorter compared to oral ADN administration. This rapid clearance of drug may not be optimal for therapeutic efficacy. The importance of a suitable formulation strategy to improve in vivo dissolution kinetics was also demonstrated here by the substantial increase in oral bioavailability achieved by ADN compared with neat BTZ043. Oral ADN was also able to achieve higher lung exposure values and showed less variation in systemic and tissue concentrations compared to neat drug. Overall, ADN formulations show substantial benefits for the improvement of BTZ043 PK. The ability to incorporate ADN into oral dosage forms, which are less expensive and more stable than inhalation formulations, may be a promising formulation strategy. The current study results merit further exploration of ADN-based oral dosage forms for BTZ043 drug delivery.

## Experimental Section

### Materials

BTZ043 (CAS Number: 1161233-85-7; 99.66% purity) was purchased from MedChemExpress (Germany). Calu-3 cells were bought from LGC Standards GmbH. Sodium dodecyl sulfate, bovine serum albumin (BSA), and ammonium acetate were from VWR. Sterile phosphate-buffered saline (PBS), Dulbecco’s modified Eagle’s medium (DMEM), fetal bovine serum (FBS), Hanks’ balanced salt solution (HBSS modified, w/calcium and magnesium, w/out phenol red), dimethyl sulfoxide (DMSO), methanol, and glucose were purchased from Sigma-Aldrich. Sterile saline (NaCl 0.9%) was purchased from B. Braun. Penicillin, streptomycin, and metoprolol tartrate were obtained from Thermo Scientific. 12-well Transwell polyester membrane cell culture inserts (Costar) are from Sarstedt. And Balb/c mice (9–11 weeks old) were bought from Envigo RMS GmbH.

### Preparation of Amorphous Drug Nanoparticles (ADN)

BTZ043 ADN was prepared via an antisolvent precipitation method as described by Rudolph et al.^24^ Briefly, 10 mg of BTZ043 (30.8 μmol) was dissolved in 1 mL of DMSO as the solvent. Sodium dodecyl sulfate (2.5 mg) and ammonium acetate (30 mg; 0.39 mmol) were dissolved in 10 mL of demineralized water as the antisolvent. The antisolvent solution was cooled to 3 °C in an ice bath, and the solvent solution (0.4 mL) was injected into the antisolvent solution under vigorous stirring (750 rpm). The mixture was also sonicated (sonication amplitude 50%; HD2070, Bandelin, Germany) during injection and for 10 s postinjection. Excess surfactant (SDS) was separated from the BTZ043 ADN dispersion by centrifugation (25,000 rpm, 20 min), and the ADN was resuspended in 0.8 mL of the antisolvent solution using sonication for 2 s to achieve a homogeneous dispersion. ADN was stored at 3–5 °C until use. The final BTZ043 concentration was 4.2 mg mL^–1^ with a drug loading of >99%.

### Characterization of Neat BTZ043 and BTZ043 ADN

Static light scattering (Mastersizer 3000 with a Hydro SV liquid dispersion unit, Malvern Panalytical, U.K.) was used to measure the size distribution of neat BTZ043. The neat BTZ043 powder was dispersed in filtered distilled water containing 2.5% Kolliphor HS15 (PEG-HS, 1 mg/mL) prior to measurement. The size distribution of BTZ043 ADN was assessed by dynamic light scattering (DLS; Malvern ZetaSizer ZS Nano; Malvern Panalytical, U.K.). Nanoparticle hydrodynamic diameters were measured on the day of in vivo administration at room temperature after 10 times dilution in phosphate-buffered saline (PBS).

### Calu-3 Permeability

The human bronchial epithelial Calu-3 cell line (ATCC; HTB-55) was used in this experiment to predict the drug permeability across the airway epithelial barrier. Cells (passage numbers 33–35) were maintained with DMEM (containing 10% FBS with penicillin-streptomycin; final concentration: 100 U mL^–1^) in a 37 °C, 5% CO_2_ incubator. Cells were seeded at a density of 2.42 × 10^5^ cells cm^–2^ on 24-well Transwell polyester membrane cell culture inserts (Costar) and cultivated for 15–16 days until the transepithelial epithelial resistance (TEER; Ω × cm^2^) reached ∼400 to 500 Ω × cm^2^. The BTZ043 ADN (4.2 mg mL^–1^, 9.7 mM) dispersion was diluted to 10 μM (4.3 μg mL^–1^) with sterile HBSS for the permeability study. The neat BTZ043 powder was first dispersed under sonication in sterile HBSS (20 mM) and then diluted to 10 μM in sterile HBSS. Additionally, neat BTZ043 was also dissolved in DMSO (20 mM) and then diluted to 10 μM (containing 0.05% DMSO) to include a solubilized positive control. Metoprolol (500 μM) was used as a high permeability^26^ reference drug.

Transport studies were performed from apical to basolateral (A to B) and from basolateral to apical (B to A) directions (*n* = 6 independent experiments per direction). For A to B studies, cells were washed twice with prewarmed 37 °C HBSS buffer and the donor or apical chamber was filled with 0.4 mL of sample in HBSS (10 μM). The basolateral acceptor compartment contained 0.6 mL HBSS supplemented with 10.8 mg mL^–1^ BSA, to generate a hydrophobic sink for BTZ043 diffusion.^32^ For B to A studies, the samples (0.6 mL) were added to the basolateral chamber, and BSA-supplemented HBSS (0.4 mL) was added to the apical chamber. Plates were then incubated at 37 °C with 100 rpm orbital shaking and 0.2 mL were removed from the basolateral (A to B) or 0.15 mL removed from the apical chamber (B to A) at time points 0, 0.5, 1, 1.5, and 2 h, after which the same volume of fresh BSA-supplemented HBSS was added to the chambers. After 2 h, the TEER was measured once again to check the monolayer integrity. BTZ043 was extracted using the method described below and quantified with HPLC-MS/MS (Ultimate 3000 UHPLC system, Thermo Fisher Scientific, San Jose, CA). The apparent permeability coefficient (*P*_app_) was then calculated with eq 2
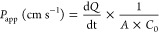
2where d*Q*/d*t* represents the steady-state flux (μmol s^–1^); *A* is the surface area of the supporter (cm^2^), and *C*_0_ is the initial concentration in the donor compartment (μM). Metoprolol concentrations were quantified via UV absorbance (Epoch2Microplate Spectrophotometer, BioTek Instruments, Inc.) at 273 nm (limit of quantification; LOQ: 0.032 μM).

### In Vivo Single-Dose Pharmacokinetics Following Oral and Intranasal Administration

All animal experiments were performed in compliance with the Guidelines for the Care and Use of Research Animals established by the 42502-2-1632 MLU – “*Bestimmung der pharmakokinetischen Parameter von Antibiotika-beladenen Nanocarriern nach intravenöser and inhalativer Gabe*”. Healthy male and female BALB/c mice (9–11 weeks old) were housed in individually ventilated cages containing filters in a specific pathogen-free environment. All of the mice had free access to food and water throughout the experiment. The animals (*n* = 6 per time point group; three male and three female) received a single-dose treatment, orally or intranasally, at five different time points (0.25, 0.5, 1, 4, and 24 h). Treatment was performed in a randomized order with *n* = 2 animals (one male and one female) from each time point treated per week for a total of 3 weeks. The oral gavage was carried out with soft sterile polypropylene gavage tubes (22G × 25 mm, FTP-22-25-50, Instech GmbH) introduced into the stomach via the esophagus. A bolus dose (200 μL; 25 mg/kg) of neat BTZ043 suspended in 1% hydroxypropyl methylcellulose (HPMC) saline solution containing 5% glucose or BTZ043 ADN in sterile saline was administered without anesthesia. For intranasal dosing (31–33), animals were anesthetized with 2.5% inhaled isoflurane (in O_2_; at 3 L min^–1^), and a nostril closed while 50 μL of BTZ043 ADN suspension diluted in sterile saline was added as a droplet into the open nostril. The position was held until the animal inhaled the droplet. After a 30 s pause in which the animal could breathe freely, the process was repeated on the other nostril (2 × 50 μL; 2.5 mg kg^–1^). After 0.25, 0.5, 1, 4, and 24 h, the animals were euthanized by CO_2_ slow flow. Blood samples were collected via cardiac puncture. Bronchoalveolar lavage was performed, and the lungs were then removed for further processing.

### Sample Collection and Processing

For plasma separation, 0.109 M sterile buffered sodium citrate (dihydrate) was freshly prepared and used as an anticoagulant. The samples were obtained after centrifugation at 1000*g* for 10 min at 4 °C. The supernatant was removed and aliquoted for both drug and urea quantification. Bronchoalveolar lung lavage fluid (BALF) was collected by introducing one catheter in the trachea and rinsing the lung three times with a total of 1.5 mL lavage fluid (sterile saline with 100 μM EDTA) and then followed by centrifugation at 800*g* for 10 min at 4 °C. The supernatant was transferred to a preweighed microcentrifuge tube, and then the weight was calculated. The lung tissue was taken after BALF collection and transferred to a preweighed centrifuge tube with three ceramic beads (zirconium oxide/yttrium stabilized, 3 mm). PBS (2-fold the lung weight in g) was added, and the tissue was homogenized in a Zentrimix 380R bead mill (Hettich, Germany) at 1500 rpm for 1.5 min. All of the samples were stored at −80 °C until use.

### Drug Extraction and Quantification

Calu-3 permeability, BAL, and plasma samples were mixed with 3× the volume of cold methanol, stored at −80 °C for 30 min to precipitate proteins, and then centrifuged at 15,000 rpm for 5 min at 4 °C. Plasma samples required a second centrifugation step to further remove all solid material. Supernatants were collected in HPLC vials and HPLC-MS/MS (Ultimate 3000 UHPLC system, Thermo Fisher Scientific, San Jose, CA) equipped with a reversed-phase column (Phenomenex, Kinetex C18, 100 Å, 1.7 μm, 2.1 mm × 150 mm), and the software Xcalibur ver. 3.1 SP3 (Thermo Fisher Scientific, San Jose, CA) was used for BTZ043 quantification. The limit of detection (LOD) and limit of quantification (LOQ) were 4 and 12 μg/L for the BALF samples and 5 and 16 μg/L for the plasma, respectively.

The concentration of urea in both BALF and plasma was determined by a urea assay kit (MAK006-1KT, Merck). Briefly, after the samples were incubated with the reaction mix for 1 h, the UV absorbance at 570 nm was measured. Since urea is considered to have an equal concentration in the capillaries and alveolar spaces, BTZ043 concentrations in the epithelial lining fluid (ELF) were calculated by using urea as the indication of the dilution factor^43^ according to eq 3(44)
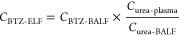
3where *C*_BTZ-BALF_ represents the BTZ043 concentration measured in the BALF and *C*_urea-plasma_ and *C*_urea-BALF_ are the urea concentrations measured from the plasma and BALF, respectively.

For comparisons of dose-normalized drug exposure in each compartment after administration via different routes, the dose-normalized relative bioavailability was calculated according to eq 4.^45^
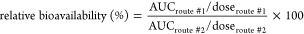
4where AUC represents the area under the curve of the concentration–time profile (μg L^–1^ h) and “dose” is defined as the nominal dose administered to animals (i.n.: 50 μg and po: 500 μg).

Drug extraction and quantification from lung homogenate was performed as follows: Aliquots of 200 μL of lung homogenate were extracted by addition of 800 μL of methanol containing (2,6-di-*t*-butyl-*p*-hydroxytoluene) BHT (0.184%) and mixed for 2 h. Afterward, 200 μL of this mixture was dried and then dissolved in 400 μL of acetonitrile. For quality control and ionization normalization, reserpine was spiked with a final concentration of 12.5 ng mL^–1^. The solution was incubated for 5 min at room temperature with continuous shaking at 1300 rpm. Afterward, 100 μL of 1% formic acid was added and the solution was incubated again under the same conditions. Samples were centrifuged (15,000*g*) for 10 min at room temperature, the supernatant was centrifuged again under the same conditions, and then 5 μL of supernatant was used for injection. The samples were analyzed by LC-MS/MS using Waters Micromass Quattro Premier XE Triple Quadrupole Mass Spectrometer (Waters Corporation, Milford, MA) coupled to an 1100 series HPLC (Agilent Technologies, Santa Clara, CA) using electrospray ionization (ESI). For LC separation, a SeQuant ZIC-HILIC column (Merck Millipore SeQuant, 2.1 inner diameter × 150 mm length with 5 μm particle size, pore-size 200 Å) with a gradient consisting of solvent A 1% formic acid and solvent B (acetonitrile) and a column temperature of 30 °C was used. LC gradient was performed. The following *m*/*z* transitions were chosen: BTZ043 (432.1 > 292.3) using a cone voltage of 30 V and a collision energy of 30 eV; reserpine (609.3 > 195.0) with 30 V and 35 eV. For each measurement, two technical replicates were performed, and measurements were performed on two separate occasions. An LOD of 0.026 and an LOQ of 0.079 μg g^–1^ were determined. All samples below the LOD were excluded from the figures and calculations.

### Statistical Analysis

All statistical analyses were performed using a two-way analysis of variance (ANOVA) with GraphPad Prism (10.0.3). The noncompartmental PK parameters, maximum concentration (*C*_max_), and area under the curve (AUC) were also determined using GraphPad Prism software.
